# Towards Efficient Detection for Small Objects via Attention-Guided Detection Network and Data Augmentation

**DOI:** 10.3390/s22197663

**Published:** 2022-10-09

**Authors:** Xiaobin Wang, Dekang Zhu, Ye Yan

**Affiliations:** Defense Innovation Institute, Chinese Academy of Military Science, Beijing 100071, China

**Keywords:** small object detection, data augmentation, image pyramid, attention mechanism, multiple detection head

## Abstract

Small object detection has always been a difficult direction in the field of object detection, especially the detection of small objects in UAV aerial images. The images captured by UAVs have the characteristics of small objects and dense objects. In order to solve these two problems, this paper improves the performance of object detection from the aspects of data and network structure. In terms of data, the data augmentation strategy and image pyramid mechanism are mainly used. The data augmentation strategy adopts the method of image division, which can greatly increase the number of small objects, making it easier for the algorithm to be fully trained during the training process. Since the object is denser, the image pyramid mechanism is used. During the training process, the divided images are up-sampled into three different sizes, and then sent to three different detectors respectively. Finally, the detection results of the three detectors are fused to obtain the final detection results. The small object itself has few pixels and few features. In order to improve the detection performance, it is necessary to use context. This paper adds attention mechanism to the yolov5 network structure, while adding a detection head to the underlying feature map to make the network structure pay more attention to small objects. By using data augmentation and improved network structure, the detection performance of small objects can be significantly improved. The experiment in this paper is carried out on the Visdrone2019 dataset and DOTA dataset. Through experimental verification, our proposed method can significantly improve the performance of small object detection.

## 1. Introduction

**Figure 1:**
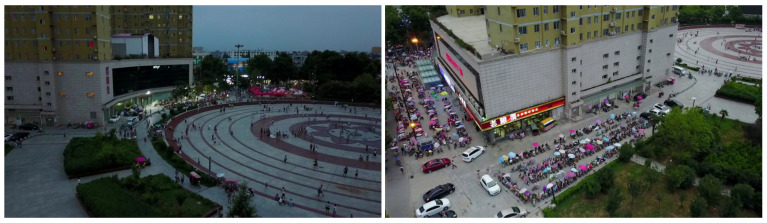
Intuitive cases about the main problems in object detection on drone-captured images.

Object detection is an important research direction in the field of computer vision, and it is the basis for other vision tasks. With the development of deep learning, object detection has been widely used, including robotics [1], autonomous driving [2], remote sensing data analysis [3], etc. Although many advanced object detection algorithms have been proposed and applied well, these advanced algorithms are not effective in object detection of UAV aerial images. The main reason is that most aerial images are small objects, and the current object detection algorithms are mainly aimed at general object detection, including large and medium-sized objects. Small object detection is still a difficult direction in the field of object detection. The current general object detection algorithm has a poor effect on small object detection, and the accuracy is usually half lower than that of large object detection [4]. This is because small object features are weak and there are few pixels. On the feature map with rich semantic information, small objects are very easy to be lost due to its low resolution, and small objects are usually dense in the image, which makes the current detection algorithm less effective in detecting small objects. Intuitive cases about the main problems in object detection on drone-captured images are shown in Figure 1.

In order to solve these two difficulties of small objects, this paper improves the performance of small object detection from the aspects of data and network structure. First of all, let us first understand what a small object is, and the definition of a small object is that the pixel below 32 × 32 is a small object according to the absolute definition method [5]. Moreover, it can be observed that the small object pixel is relatively small. From the data analysis, why it is so difficult to detect small objects. In the commonly used datasets at present, such as MScoco [5] and Pascal voc2012 [6], these datasets are mainly conventional objects with more pixels, which account for a large proportion in the image, and the objects are evenly distributed in the dataset, while small objects account for a small proportion in the dataset, and the distribution is extremely uneven. Small objects are only distributed in a small part of the image, and other images have almost no small objects. Moreover, during the training process, the number of anchor boxes matching small objects is much lower than that of large and medium-scale objects, which further causes the detection model to focus more on the detection of large and medium-scale objects and is difficult to detect small objects. Therefore, this paper uses data augmentation strategy and image pyramid mechanism to solve the problem that the number of small objects is small, and matching the small objects with anchor boxes is the difficulty. Using the data augmentation strategy to divide the original image, we take the center of each ground-truth box as the division center to divide the image, and then use non maximum suppression on the divided image to reduce too many duplicate images which reduce the training time. When the divided image is sent to the improved yolov5 for training, the divided images are upsampled into three different sizes, and then sent to the detector for detection. Finally, the detection results are fused to obtain the final detection results. Through this operation, you can make small objects larger and dense objects sparse, making it easier to match small objects with anchor boxes, thereby improving the detection performance of small objects.

In terms of the network structure, we propose an improved model Multi-Head-Attention-Yolo, which is mainly improved on the basis of Yolov5 [7]. We use two tricks, one is to add a detection head to the underlying feature map, and the other is to add an attention mechanism [8] to all the detection heads. The design of Yolov5 is still aimed at general object detection, which is not good for small object detection. Therefore, we add a detection head to the underlying feature map on the basis of Yolov5. It is known that when an image passes through the backbone network, it will go through multiple layers of downsampling. The large object will become very small after multiple layers of sampling, and after the small object has undergone multiple layers of sampling, there is almost no object information on the last layer of the feature map. Therefore, in order to improve the detection performance of small objects, we not only detect the object on the high-level feature map, but also detect the object on the low-level feature map. In this way, the detector will cover large, medium and small-size objects, which is convenient for detecting multi-size objects; Similarly, we add attention mechanism to all detection heads, so that small objects can use the context, add more semantic information, and make the detection heads pay more attention to the object.

The main contributions of this paper are as follows:

- We use the data augmentation strategy to divide images, so as to increase the number of objects more pertinently, and use non maximum suppression to reduce the number of duplicate images.
- We use the image pyramid mechanism to upsample the divided images, which can not only increase the size of small objects, but also sparse dense objects, which is convenient for dense small object detection.
- We add a detection head on the underlying feature map, so that the detector can handle multi-size objects.
- The attention mechanism is added to each detection head to make it be able to use the context, and it is easier to notice the dense small objects in the image.
- Through experimental verification, this method can significantly improve the detection performance of small objects.

The rest of the paper is organized in this way. Section 2 introduces the relevant research work of small object detection. Section 3 introduces our proposed method. Section 4 mainly introduces the datasets we use and the experimental results, and analyzes the experimental results. Section 5 is the conclusion.

## 2. Related Work

### 2.1. Data Augmentation

Data augmentation strategy is an important means to improve the performance of object detection. By using the data augmentation, the number of object can be significantly expanded, while the category remains unchanged, so that the detector is trained more fully and the detector is more robust. In early years, data augmentation strategies mainly include rotation [9], scaling, random cropping [10], translation [11], etc. These strategies have been widely used in object detection. In recent years, with the development of the object detection technology, more and more data augmentation methods have been proposed, among which the commonly used methods are CutOut [12], MixUp [13], CutMix [14] and so on. Nowadays, Yolov4 [15] and Yolov5 [7,16] use mosaic enhancement in data augmentation. Although these enhancement techniques have improved the performance of object detection, they are mainly aimed at general object detection. For the small object data augmentation strategy, Kisantal et al. [4] proposed a small object replication enhancement strategy. They found that the small object accounted for a low proportion in the dataset and the distribution was uneven. If the small object in the data set was copied to another image without a small object and cannot affect the original image object, good detection performance can be achieved through this operation. Although this operation can effectively improve the detection performance of small objects, it is time-consuming and labor-intensive for huge datasets. We use the ground-truth box center division method to quickly expand the data set, and the number of small objects will also be greatly increased. Then, the image pyramid mechanism is used for the divided image to enlarge the size of the object and make the dense small object more sparse, which is convenient for the detector to detect the small object.

### 2.2. Small Object Detection

Small object detection is not only an important research direction of object detection, but also a difficult point of object detection. Although many researchers have conducted a lot of research in this direction, the performance of small object detection is still not good. At present, researchers mainly improve the performance of small object detection from the aspects of feature fusion, contextual connection, and adversarial learning. Ref. [17] proposed an ION network. This method first clips the candidate region features from different layers of the convolutional neural network, and then normalizes the scale of the feature regions of different scales through the RoI (Region of Interest) Pooling. Finally, these multi-scale features are fused to improve the ability of the regional feature expression. FPN [18] is a widely used feature fusion method at present. It introduces a bottom-up and top-down network structure to achieve the purpose of feature enhancement by fusing the features of adjacent layers. Although feature fusion can combine shallow spatial information with deep semantic information to improve the performance of small object detection, it is inevitable that noise information will be mixed in the process of feature fusion, and feature fusion will increase the amount of computation, resulting in the failure to further improve the detection performance. Context is also an important means to improve small object detection performance. Small objects have fewer pixels and fewer features. In order to increase the amount of small object features, using context is necessary. Reference [17] proposed a global context information learning method, which transmits context information along the left, right, up and down directions on the feature map to capture valuable context. Furthermore, reference [19] proposes a spatial memory network, which achieved instance-level context modeling through multiple memory iterations. However, this method complicates the model, and the training of the model relies heavily on the initialization parameters. In terms of adversarial learning, adversarial learning is used to improve image quality and thus improve the performance of small object detection. GAN [20] network is a frequently used adversarial network. Li et al. proposed a perceptual GAN method [21] specifically for small object detection. The method learns high-resolution feature representations for small objects in a way that the generator and the discriminator play against each other. Although the adversarial network can increase the feature information of small objects and improve the detection ability of small objects, the adversarial network is very difficult to train, and it is not easy to achieve a good balance between the generator and the discriminator. It can be observed that small object detection is still full of challenges.

## 3. Method

**Figure 2:**
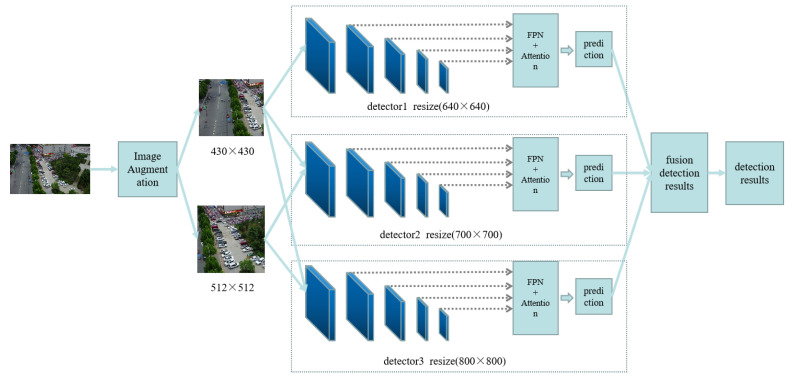
An illustration of our framework. Our network consists of two parts. One part is the data augmentation, which use the center of the ground truth object as the image division center to divide image. Moreover, it can increase the number of objects, making it easier for detectors to detect. The second part is the Multi-Head-Attention-Yolo. We add a detection header on the feature map and use the attention mechanism on each detection header.

In this section, we explain our algorithm from two aspects: data augmentation and Multi-Head-Attention-Yolo. First, we use data augmentation on the input data to increase the number of objects, making it easier for subsequent detectors to detect. Then, we use the image pyramid mechanism to make dense objects sparse and small objects larger. In order to further improve the detection performance of small objects, we proposed Multi-Head-Attention-Yolo to make it easier to detect small objects. This section is mainly composed of three parts. First, we review Yolov5 and use Yolov5 as our baseline algorithm. In the second part, we explain the data augmentation algorithm we use. The third part mainly explains our Multi-Head-Attention-Yolo. The overall network structure of this algorithm is shown in Figure 2.

### 3.1. Overview of Yolov5

Yolov5 includes the input layer, backbone network layer, neck layer, and detection head. The input layer mainly performs mosaic data enhancement, adaptive anchor box calculation, adaptive image scaling, etc., on the input image. Generally, Yolov5 uses the architecture of CSPDarknet53 with a spatial pyramid pooling(SPP) layer as backbone. The neck of Yolov5 now adopts the feature pyramid network(FPN) and path aggregation network(PAN) structure, and in the neck structure of Yolov5, the CSP2 structure designed by CSPNET is adopted to strengthen the ability of network feature fusion. In Yolov5, CIOU Loss is used as the loss function of the Bounding box. In the post-processing process of object detection, for the screening of many object boxes, non-maximum suppression (NMS) [22] operations are usually required to reduce repeated detection boxes. In Yolov5, there are four models, Yolov5s, Yolov5m, Yolov5l and Yolov5x. In this experiment, we use four model tests, respectively, and yolov5x has the highest detection accuracy. Therefore, we use Yolov5x as our baseline.

### 3.2. Data Augmentation Algorithms

The aerial image has a large field of view, the object accounts for a small proportion in the whole image, and the object is dense. In order to detect the object more accurately, we use the data augmentation strategy to divide the image, so as to separate the foreground from the background, better detect the object, and significantly reduce the training time of the detector. According to the dataset annotation file, we make statistics on the dataset. The statistical range of the dataset is 0–8, 8–16, 16–32, 32–64, 64–128, 128–256, 256–512, 512–. According to the statistics, the number of object pixels is mainly concentrated between 16 and 128 pixels. Our partition strategy is as follows, taking 32 pixels as the boundary, objects below 32 pixels are divided into 430 × 430, and objects above 32 pixels are divided into 512 × 512. When dividing aerial images, the center of the ground-truth object is taken as the image division center, and the image is divided into as many images as there are objects in it. The size of the divided image is determined according to the size of the ground-truth object. If the original image is insufficient to divide the size when dividing the image, the average value of the dataset pixel is used as the filling pixel to enlarge the image to reach the divided image size. After obtaining the divided image, we use the NMS [22] strategy to reduce the divided image. Because the objects in the aerial image are generally clustered, the images divided by the center division will have a lot of overlap, resulting in an increase in the amount of data, which increases the amount of computation. After the NMS, the image repetition will be reduced, and the amount of calculation will be reduced, accordingly.

### 3.3. Image Pyramid Mechanism

After obtaining the divided images, we use the divided images to train Multi-Head-Attention-Yolo. Although the divided images reduce the background information, the objects are still very dense, and the detection is still difficult. In order to improve the detection performance, we use the image pyramid mechanism. The Multi-Head-Attention-Yolo algorithm will first sample the input image, and then send it to the detector for detection. We upsample our divided images to 640 × 640, 700 × 700, 800 × 800, and then they are respectively sent to three detectors, and the detection results of the three detectors are fused, and finally the detection results are obtained.

### 3.4. Multi-Head-Attention-Yolo

**Figure 3:**
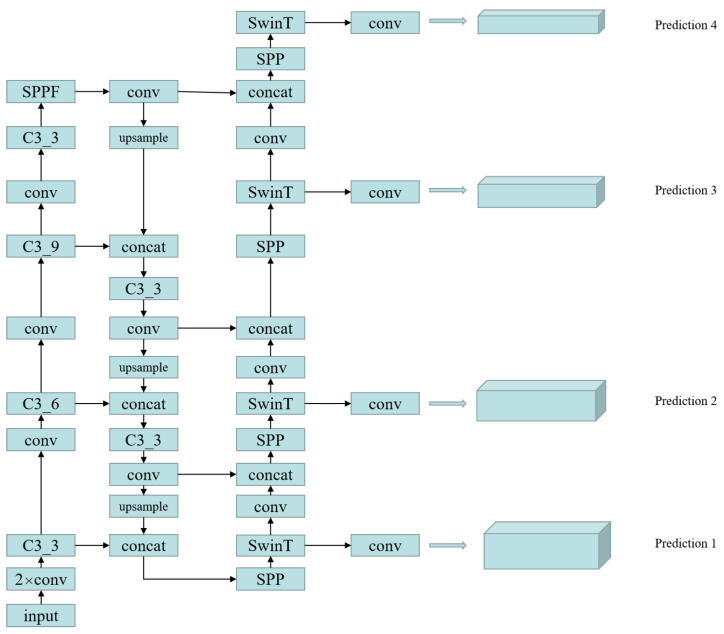
Detailed network structure diagram of Multi-Head-Attention-Yolo. We add a detection header on the low-level feature map and use the attention mechanism on detection header. Where C3_X represents 3 convs and X bottleneck and SwinT represents swin transformer.

**Figure 4:**
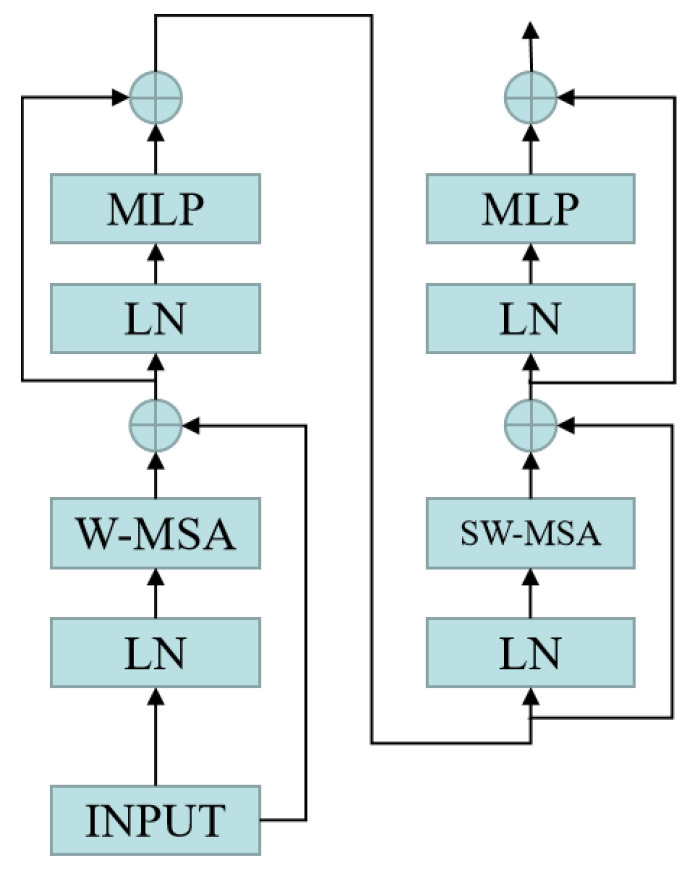
The architecture diagram of swin transformer.

Multi-Head-Attention-Yolo is improved on Yolov5. The detailed network structure diagram of Multi-Head-Attention-Yolo is demonstrated in Figure 3. However, Yolov5 is designed to detect general objects. Yolov5 is not effective for small object detection. The main reason is that it mainly designs detection heads on high-level feature maps. It is mainly aimed at medium and large objects, while small objects are mainly concentrated on the low-level feature map, and small objects have almost no semantic information on the high-level feature map. Therefore, in order to improve the detection performance of small objects, we add a detection header on the P2 feature map on the basis of Yolov5, so that there are detection headers on P2, P3, P4 and P5 that can cover most objects. At the same time, we replace some convolutional blocks and CSP bottleneck blocks in the original version of YOLOv5 with swin transformer blocks [23,24] on each detection header, which can make the detector pay more attention to the region of interest and can use the context to increase the feature information of the small object, so as to further improve the detection performance of the small object. A swin transformer block consists of a shifted window-based multi-head self attention(MSA) module, followed by a 2-layer multilayer perceptron(MLP) with GELU nonlinearity in between. A LayerNorm (LN) layer is applied before each MSA module and each MLP, and a residual connection is applied after each module. Figure 4 shows the architecture diagram of the swin transformer.

## 4. Experiments

### 4.1. Experimental Setting

Dataset. This experiment is mainly verified on the Visdrone2019 [25] and DOTA [26] datasets. The Visdrone2019 dataset includes 6471 images as the train set, 548 images as the validation set, and 1610 images as the test set. Dota dataset are satellite remote sensing images with a total of 16 categories. There are 1411 images in the train set and 458 images in the validation set. The image resolution ranges from 800 × 800 to 4000 × 4000. The objects in the dataset are mostly small objects.

Implementation details. We implement the proposed approach using the pytorch in this paper, and use NVIDIA 3090 for training and testing. We use the Yolov5x pre-training model to initialize our model before training. Because the original structure of Yolov5 has changed, we only select the part we use to initialize our model. We train 60 epochs in the whole network structure and use the Adam optimizer to train and optimize the network. The initial learning rate is 0.001. By the time of the last epoch, the learning rate will be 0.12 times the original. The batch size is set to 8, because we augment the original image to make the size smaller. During training, we can input multiple images at a time, which is more conducive to the detection of small objects.

Evaluation Metrics. We use commonly used detection metrics, such as mAP, AP50, $AP_{s}$, $AP_{m}$, $AP_{l}$, AR100 as our evaluation metrics. Specifically, mAP is computed by averaging over all 10 intersection over union(IoU) thresholds (i.e., [0.5:0.05:0.95]) of all object categories. AP50 is computed at the single IoU threshold 0.5 over all categories. $AP_{s}$ is computed by averaging over all 10 IoU thresholds of all small object categories. Similarly, $AP_{m}$ and $AP_{l}$ are the average precision of medium object categories and large object categories, respectively. Moreover, AR100 is the maximum recall given 100 detections per image. Detailed evaluation metrics refer to [5].

### 4.2. Results

Our experiments are mainly carried out on the Visdrone2019 and DOTA datasets. In order to verify the effectiveness of our proposed method, we compare it with baseline Yolov5. The experimental results are shown in Table 1. The baseline becomes 40.3 overall *AP* and 32.5 $AP_{s}$ for small objects. Moreover, our approach achieves 41.8 *mAP* and 33.7 $AP_{s}$. We can observe that our proposed method is 1.5 higher than the baseline Yolov5 about *mAP* and $AP_{s}$ improved by 1.2. Experiments demonstrate that our proposed method can significantly improve the detection performance of small objects. At the same time, we compare it with other algorithms, including two-stage algorithms, Faster R-CNN [27], Swin transformer Mask R-CNN [24], etc. It can be observed that our algorithm is still the best when the same data set is used (including data augmentation), and the performance of small object detection is the highest.

To prove that the data augmentation strategy is effective, we made a comparison with Yolov5 and TPH-yolov5 [23]. The experimental results are shown in Table 2. Because our method uses data augmentation, the detection performance is significantly better than that of algorithms without using data augmentation. The detection accuracy of different object categories is shown in Table 3. From the table, it can be observed that the car has the highest detection accuracy, while the awning-tricycle detection accuracy is the lowest. This is because different objects account for different proportions in the data set. The car has a large amount of object, which is easier to train during training, while the awning-tricycle has a small proportion in the whole data set, which is difficult to train during training, so the detection accuracy is low.

Similarly, we tested our proposed algorithm on the DOTA data set, and the experimental results are shown in Table 4. It can be observed that, compared with other algorithms, our proposed algorithm still has the highest detection accuracy, and the small object detection accuracy is the highest.

**Figure 5:**
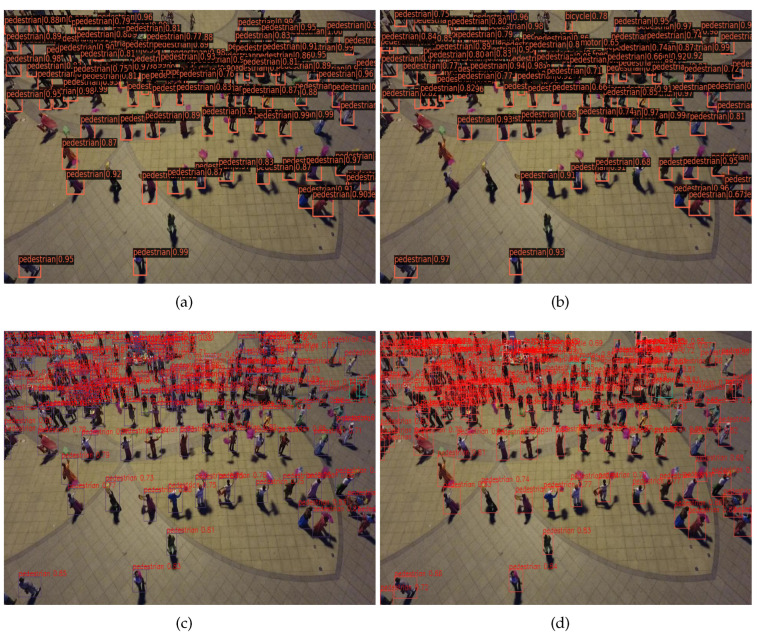
Visualization results of four algorithms. (**a**) Faster R-CNN (**b**) Swin transformer Mask R-CNN (**c**) Yolov5 (**d**) Ours.

Our methods have a good detection performance, because we have used data augmentation, image pyramids, and Multi-Head-Attention-Yolo, including the use of attention mechanisms and multiple detection heads. Data augmentation can increase the number of objects. The increase in the number of objects makes the anchor box easier to match with the ground truth, which makes the detector more fully trained. At the same time, we use the image pyramid mechanism for the divided image, which can make small objects larger and dense objects sparse, thus improving the accuracy of image detection. In the Multi-Head-Attention-Yolo, we use the swin transformer block to replace part of the convolution layer of detection head. The swin transformer block can capture global information and abundant contextual information. It can also enhance the feature representation with self-attention mechanism. Moreover, multiple detection heads can increase the feature information of each layer, because the combination of high-level semantic features and low-level spatial features can enrich the feature information and facilitate the detection of small objects. Therefore, our method has a good detection performance. Figure 5 shows the detection results of the different methods.

### 4.3. Ablation Studies

#### 4.3.1. Effect of Data Augmentation

In order to verify that our data augmentation strategy can improve the detection performance of small objects, this experiment uses the algorithm with data augmentation strategy and image pyramid mechanism to compare with the algorithm without the data augmentation strategy and image pyramid. All experiments were performed on the baseline Yolov5, and Yolov5 did not improve. We conduct experiments on the VisDrone2019. For Yolov5 without data augmentation strategy, we resize the input image to 1536 pixels. Because the input images are large, we only input two images at a time during training. Other setting parameters are the same. The experimental results are shown in Table 5. It can be observed from the table that using our data augmentation strategy is better than without data augmentation strategies, improving the mAP and $AP_{s}$ by 3.2 and 4.4. We use the center of each object to divide the image. Each object has a corresponding divided image, which greatly increases the number of objects. At the same time, we also use the image pyramid mechanism to upsample the divided images, making small objects larger and dense objects sparse. Therefore, our strategy has a high detection performance.

#### 4.3.2. Effect of Multiple Detection Heads

Yolov5 is mainly designed to detect general objects, so only P3, P4 and P5 are used in the feature pyramid, and three detection heads are set on the P3, P4 and P5 feature maps. Our main purpose is to solve small object detection. After convolution and pooling, there is almost no information on the high-level feature map. Therefore, in order to improve the performance of small object detection, we also added detection heads to the bottom feature map of Yolov5 to detect objects on the four feature maps P2, P3, P4 and P5. The experimental results are shown in Table 5. Compared with baseline Yolov5, we can observe that our proposed method can significantly improve the detection performance of small objects by using multiple detection heads.

#### 4.3.3. Effect of Attention Mechanism

The attention mechanism is to make the network pay more attention to the object area, and the attention mechanism can contact the context to enrich the feature information of small objects. This paper improves the Yolov5 on the basis of Yolov5, replacing the convolution part of all detection heads with swin transformer blocks. The experimental results are shown in Table 5. Compared with the Yolov5 without improvement, we can observe that the detection accuracy of objects has been improved by modifying the detection heads.

## 5. Conclusions

In this paper, we use data augmentation strategy, image pyramid mechanism, and improvements to Yolov5, using the swin transformer to replace the convolutional layer of the detection head, and adding multiple detection heads to Yolov5. By using these techniques, we have experimented on Visdrone2019 and DOTA datasets, respectively. After experimental verification, our proposed method can significantly improve the detection accuracy of small objects. Next, we will continue to research on small object detection, hoping to improve the detection performance of small objects while reducing the amount of computation.

## Figures and Tables

**Table 1:** Experimental results of each algorithm on the Visdrone2019 validation set.

Method	mAP	AP50	${\mathrm{AP}}_{s}$	${\mathrm{AP}}_{m}$	${\mathrm{AP}}_{l}$	AR100
Faster R-CNN	34.1	56.7	27.2	46.9	43.2	47.5
Swin transformer Mask R-CNN	37.5	59.9	30.9	50.0	51.4	50.3
Yolov5	40.3	64.1	32.5	53.3	58.2	58.5
Ours	41.8	66.1	33.7	54.2	59.4	59.2

**Table 2:** The comparison of the performance in VisDrone2019 validation set about using and not using data augmentation.

Method	mAP	AP50	AP75
Yolov5 + no data augmentation	36.8	58.5	38.9
TPH-Yolov5 + no data augmentation	38.3	60.3	40.8
Ours	41.8	66.1	43.6

**Table 3:** Detection performance of each object category on the Visdrone2019 validation set.

Method	Pedes-Trian	People	Bicycle	Car	Van	Truck	Tricycle	Awning-Tricycle	Bus	Motor
Yolov5 + no data augmentation	35.9	24.8	21.3	65.3	43.8	39.1	29.9	17.9	56.9	33.6
TPH-Yolov5 + no data augmentation	39.2	27.1	23.3	68.1	45.4	39.6	29.5	18.4	56.3	36.1
Ours	41.4	29.5	25.3	68.1	49.9	43.1	35.2	21.9	61.5	39.3

**Table 4:** Experimental results of each algorithm on the DOTA validation set.

Method	mAP	AP50	${\mathrm{AP}}_{s}$	${\mathrm{AP}}_{m}$	${\mathrm{AP}}_{l}$	AR100
Faster R-CNN	44.1	71.0	35.7	49.3	46.4	53.8
Swin transformer Mask R-CNN	46.9	72.9	36.8	51.4	52.4	56.8
Yolov5	49.8	75.6	37.0	52.9	54.9	61.1
Ours	50.7	76.9	37.8	53.4	54.7	61.5

**Table 5:** Ablation studies on the Visdrone2019 validation set.

Method	mAP	AP50	${\mathrm{AP}}_{s}$	${\mathrm{AP}}_{m}$	${\mathrm{AP}}_{l}$
Yolov5	36.8	58.5	28.1	49.7	59.7
Yolov5 + data augmentation + image pyramid	40.3	64.1	32.5	53.3	58.2
Yolov5 + previous + multiple detection head	41.0	65.0	33.4	53.3	57.0
Yolov5 + previous + attention	41.8	66.1	33.7	54.2	59.4

## Data Availability

The data used in this study are available on request from the corresponding author.
